# Ionic Liquid-Based Extraction Strategy for the Efficient and Selective Recovery of Scandium

**DOI:** 10.3390/molecules29174007

**Published:** 2024-08-24

**Authors:** Sheli Zhang, Yuerong Yan, Qiang Zhou, Yunchang Fan

**Affiliations:** 1 School of Science and Technology, Jiaozuo Teachers College, Jiaozuo 454000, China; 1295006035@jzsz.edu.cn (S.Z.); 1295002321@jzsz.edu.cn (Y.Y.); 2 College of Chemistry and Chemical Engineering, Henan Polytechnic University, Jiaozuo 454003, China; 212112010027@home.hpu.edu.cn

**Keywords:** ionic liquids (ILs), phenylphosphinic acid (PPAH), extraction, scandium (Sc)

## Abstract

The recovery of scandium (Sc) from highly acidic industrial effluents is currently hindered by the use of large quantities of flammable and toxic organic solvents. This study developed an extraction system using ionic liquids (ILs) and phenylphosphinic acid (PPAH) as diluents and an extractant, respectively, to selectively recover Sc from the aqueous phase. The effect of IL chemical structure, aqueous pH and temperature on the extraction of Sc was systematically investigated and the findings revealed that ILs with longer alkyl side chains had reduced Sc extraction ability due to the presence of continuous nonpolar domains formed by the self-aggregation of the IL alkyl side chain. The IL/PPAH system maintained high extraction ability toward Sc across a wide temperature range (288 K to 318 K) and the extraction efficiency of Sc could be improved significantly by increasing the aqueous pH. The extraction process involved proton exchange, resulting in the formation of a metal−ligand complex (Sc(PPA)_3_).

## 1. Introduction

The rare earth element Sc and its compounds are essential in various industries like fuel cells [1], alloys [2], and nuclear sectors [3,4,5]. Scandium is found in mineral ores like bauxite, rutile and ilmenite ores at low concentrations (10–330 mg kg^−1^) [6,7] and is typically extracted as a byproduct during the production of other metals [7,8]. Currently, methods such as adsorption [9], nanofiltration [10] and solvent extraction (SE) [7,8] are used to recover and purify scandium. Solvent extraction is the most commonly used method, which typically involves using a combination of di-(2-ethylhexyl) phosphate (D2EHPA) and tributyl phosphate (TBP) as extractants, along with sulfonated kerosene as a diluent [8,11]. However, the use of volatile and flammable organic solvents like sulfonated kerosene in SE can pose environmental risks, highlighting the need to develop eco-friendly solvents for scandium recovery.

Ionic liquids (ILs) entirely composed of ions are regarded as new types of eco-friendly solvents due to their negligible vapor pressure, low flammability and high chemical stability. They have been utilized in the extraction of Sc [12,13,14]. Depuydt and colleagues created an aqueous biphasic system (ABS) composed of a carboxyl-functionalized phosphonium IL ([P_444_C_1_COOH]Cl) and a 16 wt% NaCl solution to extract Sc. This system exhibited excellent performance for the extraction of Sc via the coordination of the carboxylate group of [P_444_C_1_COOH]Cl with Sc(III) [12]. Chen and coworkers also synthesized a series of hydrophobic carboxyl-functionalized ILs, 1-alkylcarboxylic acid-3-methylimidazolium bis(trifluoromethanesulfonyl)imide ([(CH_2_)*_n_* COOHmim][NTf_2_]) (*n* = 3, 5, 7) to act as extractants to recover Sc from the water phase. It was observed that the extraction efficiency (*EE*) of Sc increased upon increasing the aqueous phase pH value, achieving an extraction efficiency as high as 99.5% at pH 4.2 [13]. Zhang et al. constructed a homogeneous liquid–liquid extraction system (HLLES) consisting of carboxylic acid-functionalized ILs (*N*-carboxymethylpyridinium bis(trifluoromethylsulfonyl)imide ([HbetPy][NTf_2_]) and *N*-carboxymethyl-*N*-methylmorpholinium bis(trifluoromethylsulfonyl)imide ([HbetMor][NTf_2_])) and water. This system transformed into a uniform solution at 342 K ([HbetPy][NTf_2_]) or 347 K ([HbetMor][NTf_2_]) during extraction and reverted to a two-phase system upon cooling to room temperature. In comparison to traditional two-phase systems, HLLES demonstrated enhanced extraction efficiency for Sc (with an equilibrium extraction efficiency of 94.42% at 342 K) [14].

The above findings indicate that ILs show promise as alternatives to traditional solvents for the extraction of Sc.

However, there are challenges to address when using carboxyl-functionalized ILs as extractants. Scandium ions are transferred to IL phases through the formation of Sc-carboxylate complexes. Yet, the pKa values of carboxyl groups in fatty acids are typically around 4.7 [15,16]. This means that at a pH below 4.7, carboxylates can become protonated, weakening the coordination of Sc-carboxylate complexes and significantly reducing Sc extraction efficiency. For instance, the extraction efficiencies of certain carboxyl-functionalized ILs towards Sc are very low at pH levels below 3 [13,14]. This limitation hinders their use in extracting Sc from highly acidic environments, such as titanium dioxide waste acid (TDWA) with a pH of around −0.7 to 0, which is a common source of Sc [17,18].

Although the ABS made up of [P_444_C_1_COOH]Cl and NaCl demonstrates a high ability to extract Sc even at a pH of −0.3, it requires 16% NaCl to form the ABS. However, the solubility of the IL cation exceeds 10% at an aqueous pH of around 0 [12], leading to challenges in disposing of the added NaCl and the dissolved IL cation in industrial settings. Therefore, developing a hydrophobic IL system for efficient Sc extraction under high acidity is crucial. This study proposes an extraction system based on hydrophobic ILs using phenylphosphinic acid (PPAH) as the extractant to recover Sc from the water phase. PPAH was chosen due to its strong acidity (pK_a_ = 1.75 [19]) and its ability to function as an extractant in highly acidic conditions.

## 2. Results and Discussion

### 2.1. Screening of ILs

**Figure 1:**
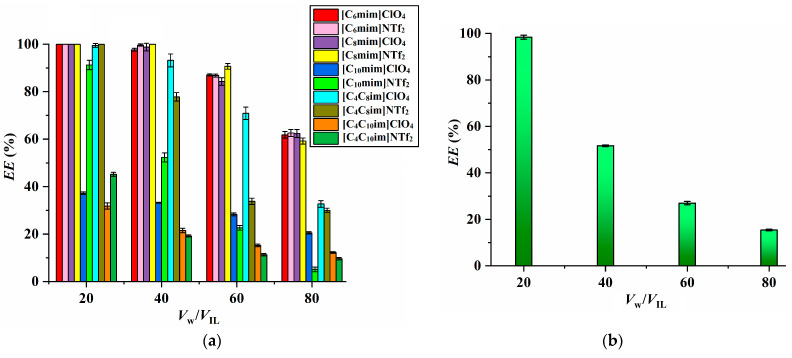
Extraction ability of different IL/PPAH systems (**a**) (Sc concentration, 20 mg L^−1^; extraction time, 10 min; extraction temperature, 298 K; pH, 2.0; PPAH concentration in each IL, 10 g L^−1^) and that of [C_4_C_8_im]ClO_4_/PPAH system (**b**) (PPAH concentration in [C_4_C_8_im] ClO_4_, 21.6 g L^−1^). The volume ratio of water phase to IL phase is noted as *V*_w_/*V*_IL_.

In this work, ten ILs including 1-alkyl-3-methylimidazolium ([C*_n_*mim]X, *n* = 6, 8, 10, X = ClO_4_^−^, NTf_2_^−^) and 1-alkyl-3-butylimidazolium ([C_4_C*_n_*mim]X, *n* = 8, 10, X = ClO_4_^−^, NTf_2_^−^) were used as extraction solvents to recover Sc from water. The results depicted in Figure 1a indicate that the extraction efficiency (*EE*) of Sc is sensitive to the length of the alkyl chain in the IL cation: it remains relatively stable for the ILs with shorter alkyl chains ([C_6_mim]X and [C_8_mim]X; X = ClO_4_^−^, NTf_2_^−^) but decreases remarkably when the alkyl chain length is increased to decyl ([C_10_mim]X; X = ClO_4_^−^, NTf_2_^−^). This trend is likely due to the tendency of alkyl side chains in the IL cations to cluster together, forming nonpolar regions. As the length of the alkyl side chain increases, these nonpolar regions also increase [20,21,22], which hinders the transfer of the polar scandium-phenylphosphinate complex (Sc(PPA)_3_) from the water phase to the IL phase during extraction.

Generally, the extraction ability of the ILs toward metal ions is significantly affected by their physicochemical properties such as viscosity, hydrophobicity and polarity [13,23,24,25]: ILs with low viscosity are more efficient for the extraction of metal ions because low viscosity is beneficial to the mass transport of metal ions in the extraction process. Additionally, in the extraction of metal ions, it is commonly thought that the metal ions form complexes with ligands (extractants) to create hydrophobic metal–ligand complexes, which then tend to enter the highly hydrophobic and polar IL phases. It should be noted that the solubilities of ILs are closely related to their hydrophobicity [26,27]: ILs with lower solubilities are more hydrophobic and solubility is thus used as an indicator of the hydrophobicity of the ILs, while the ILs with lower *E*_NR_ values (molar transition energies of Nile Red (NR)) are considered more polar [28,29,30].

The data listed in Table 1 indicate that there is no apparent correlation between the physical characteristics of ionic liquids (ILs), such as viscosity, hydrophobicity and polarity, and their effectiveness in extracting scandium. For instance, despite [C_6_mim]NTf_2_ having a lower viscosity, higher hydrophobicity, and lower polarity compared to [C_6_mim] ClO_4_, its ability to extract scandium is similar to that of [C_6_mim]ClO_4_.

Additionally, the data presented in Table 1 show that the solubility of PPAH in ClO_4_-based ILs is greater than that in NTf_2_-based ILs.

To further explore whether increasing the concentration of PPAH in ILs can enhance the *EE* values of Sc, additional experiments were conducted using PPAH-saturated [C_4_C_8_im]ClO_4_ (with a PPAH concentration of 21.6 g L^−1^) for Sc extraction. The results depicted in Figure 1b indicate that the extraction efficiency of the PPAH-saturated [C_4_C_8_im]ClO_4_ is lower compared to that of [C_4_C_8_im]ClO_4_ with a lower PPAH concentration (10 g L^−1^). This difference could be attributed to the “common-ion effect”, where the high concentration of PPAH hinders the transfer of Sc(PPA)_3_ from water to the IL phase due to the presence of the same anion PPA^−^ [35,36].

Finally, in view of the fact that the four ILs, [C_6_mim]ClO_4_, [C_6_mim]NTf_2_, [C_8_mim] ClO_4_ and [C_8_mim]NTf_2_ exhibit comparable extraction abilities toward Sc (Figure 1a) but [C_8_mim]NTf_2_ has the lowest water solubility according to Table 1, it is chosen as the preferred solvent for extraction and is utilized in subsequent experiments.

### 2.2. Effect of the PPAH Concentration, Temperature, Extraction Time, Initial pH Value of Aqueous Phase and Sc Concentration

**Figure 2:**
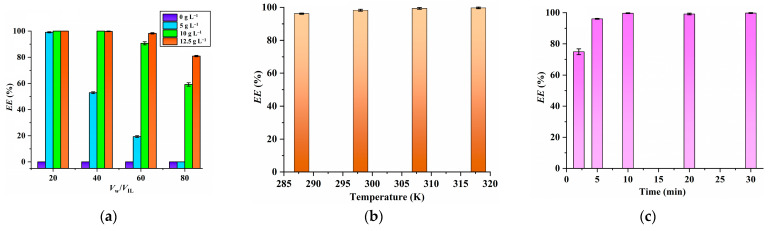
Effects of PPAH concentration (**a**) (Sc concentration, 20 mg L^−1^; extraction time, 10 min; extraction temperature, 298 K; pH, 2.0), extraction temperature (**b**) (Sc concentration, 20 mg L^−1^; extraction time, 10 min; pH, 2.0; PPAH concentration in the IL, 12.5 g L^−1^) and extraction time (**c**) (Sc concentration, 20 mg L^−1^; pH, 2.0; PPAH concentration in the IL, 12.5 g L^−1^; extraction temperature, 298 K) on the extraction of Sc.

In this work, the effects of PPAH concentration, temperature, initial pH value of aqueous phase and Sc concentration on the extraction of Sc were investigated. The results illustrated in Figure 2a suggest that the *EE* values of [C_8_mim] NTf_2_ toward Sc increase as the PPAH concentration rises. It is important to highlight that the *EE* value of [C_8_mim] NTf_2_ without PPAH is zero, meaning that the extraction ability of pure IL toward Sc is poor. Since PPAH-saturated [C_8_mim]NTf_2_ (PPAH concentration, 12.5 g L^−1^) has the highest EE values, it is selected for further experiments.

As can be seen from Figure 2b, the *EE* values always remain above 96% in the temperature range of 288 K to 318 K, suggesting that the extraction of Sc by the [C_8_mim] NTf_2_/PPAH system can be operated over a broad temperature range. The results illustrated in Figure 2c suggest that the extraction can reach equilibrium within 10 min, highlighting the rapid extraction rate of the proposed [C_8_mim]NTf_2_/PPAH system.

**Figure 3:**
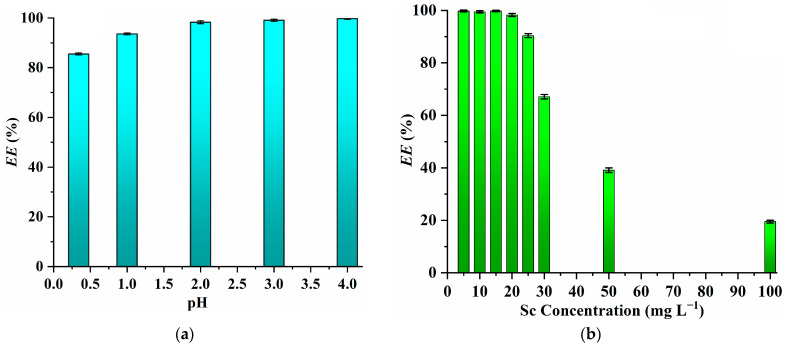
Effect of aqueous pH (**a**) (Sc concentration, 20 mg L^−1^; extraction time, 10 min; extraction temperature, 298 K; PPAH concentration in the IL, 12.5 g L^−1^) and Sc concentration (**b**) (extraction time, 10 min; extraction temperature, 298 K; PPAH concentration in the IL, 12.5 g L^−1^; pH, 2.0) on the extraction of Sc.

Additionally, the *EE* values almost remain constant in the range of pH 4.0 to 2.0 but decrease as the initial pH of the aqueous phase decreases further (Figure 3a). This trend can be understood through the following equation:

(Sc^3+^)_w_ + 3(PPAH)_IL_ ↔ (Sc(PPA)_3_)_IL_ + 3(H^+^)_w_ (1)

where the subscript letters w and IL refer to the water and IL phases, respectively.

Clearly, if the concentration of acid is increased, the balance of extraction will move towards the left, thereby decreasing the ability to extract Sc^3+^.

Additionally, it is evident from Figure 3b that the *EE* values remain consistent when the Sc concentration ranges from 5 mg L^−1^ to 20 mg L^−1^ but decreases notably as the Sc concentration increases beyond this range. This decline can be attributed to the need for a higher amount of PPAH to form Sc(PPA)_3_ at higher Sc concentrations, without any additional PPAH being available at the same IL dosage.

Finally, the effect of anion on the extraction was also studied and the experimental findings revealed that the *EE* value for Sc_2_(SO_4_)_3_ (20 mg L^−1^ of Sc^3+^, pH 2.0; *V*_w_: *V*_IL_, 20:1; PPAH concentration in [C_8_mim]NTf_2_, 12.5 g L^−1^) is 99.9%, nearly identical to that of ScCl_3_ (100%). This implies that the extraction is unaffected by the type of anions present in scandium salts.

### 2.3. Extraction Mechanism

As described in Equation (1), Sc^3+^ was combined with the PPAH present in the IL phase to form Sc(PPA)_3_ during the extraction process. To verify this hypothesis, following the extraction, the IL-PPAH mixture was combined with ethanol to dissolve the IL, resulting in the production of a white solid. This solid was dissolved using a 2 mol L^−1^ HCl solution, and the concentrations of Sc and PPAH in the HCl solution were determined using UV-Vis spectroscopy (see Section 3.3 and Section 3.4). The analysis reveals that the ratio of Sc to PPAH molecules is 1:2.9, which closely matches the expected stoichiometric ratio of 1:3.

Moreover, Equation (2) describes the interaction between Sc^3+^ and phenylphosphinate, while Equation (3) expresses the stability constant (K) of the complex Sc (PPA)_3_. The K value is determined to be 4.1 × 10^13^ (L mol^−1^)^4^. It is known that PPAH is a weak acid with a dissociation constant (K_a_) of 10^−1.75^ (Equations (4) and (5)) [19]. As a result, pH affects the stability of the complex Sc(PPA)_3_, and Equation (8), obtained via combining Equations (3) and (5)–(7), represents the conditional stability constant (K’). In OTHER words, when the aqueous phase’s acidity increases, the complex’s stability decreases.

Sc^3+^ + 3PPA^−^ ↔ Sc (PPA)_3 (s)_ (2)

(3) $$K\;=\frac{1}{{[\mathrm{Sc}}^{3+}{][\mathrm{PPA}}^{-}{]}^{3}}$$

PPAH ↔ PPA^−^ + H^+^ (4)

(5) $$\mathrm{Ka}\;=\frac{{[\mathrm{PPA}}^{-}{]\;[H}^{+}]}{\left[\mathrm{PPAH}\right]}$$

[PPA’] = [PPA^−^] + [PPAH] (6)

(7) $$K^{\prime }=\frac{1}{{[\mathrm{Sc}}^{3+}][{\mathrm{PPA}}^{\prime }{]}^{3}}$$

(8) $$\log K^{\prime }\;=\;\log\;K-3\log\;(1+\frac{{[H}^{+}]}{\mathrm{Ka}})$$

where the subscript letter, s, indicates a solid state.

**Figure 4:**
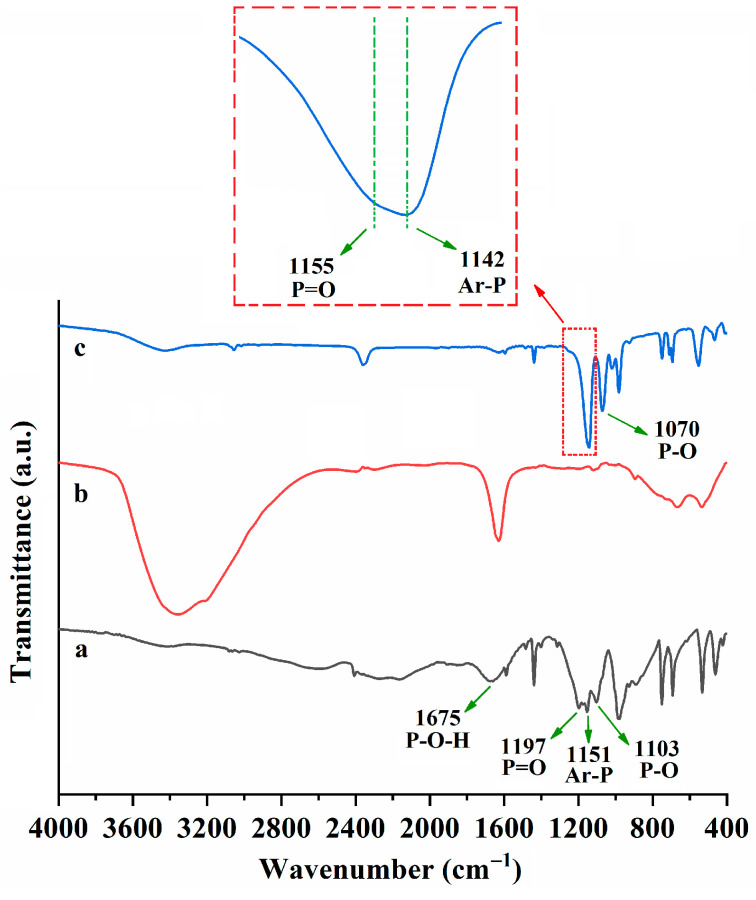
The FT-IR spectra of PPAH (**a**), ScCl_3_·6H_2_O (**b**) and Sc (PPA)_3_ isolated from [C_8_mim] NTf_2_/PPAH system after extraction (**c**).

To further prove the extraction mechanism, the Fourier transform infrared (FT-IR) spectra of PPAH, ScCl_3_·6H_2_O and the resulting white precipitate (Sc(PPA)_3_) obtained from the IL/PPAH system post extraction were measured. As shown in Figure 4, the characteristic absorption peaks of PPAH appear at 1675 cm^−1^ (the bending vibration of P–OH), 1197 cm^−1^ (the stretching vibration of P=O), 1151 cm^−1^ (the stretching vibration of phenylphosphinate P–C (Ar–P)) and 1103 cm^−1^ (the stretching vibration of P–O), respectively [37,38,39]. After extraction, the disappearance of the bending vibration of P–OH suggests that the hydrogen ion of the P–OH group is replaced by Sc^3+^ during extraction. Additionally, the stretching vibration frequencies of P=O, Ar–P and P–O all decrease post extraction (Figure 4), further confirming the interaction between Sc^3+^ and the P–OH group of PPAH [37,38,39].

Finally, the enthalpy change (∆*H*, kJ mol^−1^) during the extraction process was calculated using the following equations [13,37]:

lg *D* = − ∆*H*/(2.303R*T*) + C (9)

*D* = *C*_IL_/*C*_w_ (10)

where *D*, R and C refer to the distribution ratio, gas constant and a constant; *C*_w_ and *C*_IL_ denote the Sc concentrations in the aqueous and IL phases, respectively.

**Figure 5:**
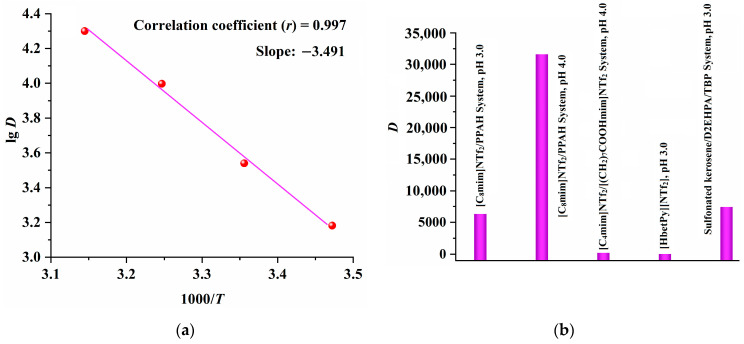
The relationship curve between lg*D* and 1000/*T* (**a**) and comparison of the [C_8_mim] NTf_2_/PPAH with the reported extraction systems (**b**).

The curve of lg *D* versus 1000/*T*, shown in Figure 5a, suggests that the ∆*H* value is positive (66.8 kJ mol^−1^), indicating that the extraction of Sc by the IL/PPAH system is an endothermic process [13,37].

### 2.4. Comparison of [C_8_mim]NTf_2_/PPAH with the Reported Extraction Systems

Recently, IL-based extraction systems such as the 1-butyl-3-methylimidazolium bis(trifluoromethylsulfonyl)imide ([C_4_mim]NTf_2_)/[(CH_2_)_7_COOHmim][NTf_2_] [13] and [HbetPy][NTf_2_] [14] systems, as well as the traditional extraction system of sulfonated kerosene (SK)/D2EHPA/TBP [8,11] have been effectively used for the recovery of Sc from the water phase. Therefore, the extraction ability of the proposed [C_8_mim]NTf_2_/PPAH system toward Sc is compared with the reported methods and the results depicted in Figure 5b suggest that the extraction ability of [C_8_mim]NTf_2_/PPAH (*D* value, 6.3 × 10^3^, pH 3.0) surpasses that of the [C_4_mim]NTf_2_/[(CH_2_)_7_COOHmim] [NTf_2_] and [HbetPy][NTf_2_] systems and is close to that of SK (85%)/D2EHPA (10%)/TBP (5%).

Furthermore, it has been observed that PPAH has relatively low levels of toxicity and does not cause any adverse effects when fed to rats for 28 days at a daily dose of up to 859 mg kg^−1^ [40], even if the median lethal dose (LD_50_) value is unknown. Both D2EHPA and TBP have modest toxicity levels (LD_50_s of 4.9 g kg^−1^ [41] for D2EHPA and 1.863 g kg^−1^ [42] for TBP). The dissolution losses of [C_8_mim]NTf_2_ and PPAH are both 3.0% [32] (*V*_w_:*V*_IL_ = 40:1) during the extraction process. The dissolution losses of D2EHPA and TBP [11] are about 0.55% [43] and 2.1% [44], respectively, under the same conditions. In terms of cost, PPAH, D2EHPA and TBP are 1067.85 RMB (500 g; purity, 99%), 2296.39 RMB (500 g; purity, 97%) and 3055.49 RMB (500 mL; purity, ≥99%), respectively [45]. Finally, using the [C_8_mim]NTf_2_/PPAH extraction system, Sc is extracted as Sc(PPA)_3_ and this complex can be calcined [46] to produce scandium phosphate, an electrolyte for solid-state batteries [47].

Overall, PPAH exhibits a similar extraction performance (*D* value) towards Sc^3+^, lower cost and larger dissolution loss compared to the traditional extractant D2EHPA/TBP. Furthermore, D2EHPA/TBP can be reused via back extraction and PPAH can be converted into Sc(PPA)_3_, meaning that the consumption of PPAH is higher than D2EHPA/TBP. The development of IL-based extraction systems that make it easy to recycle PPAH may be the direction of future work, which is currently underway.

### 2.5. Practical Application

**Figure 6:**
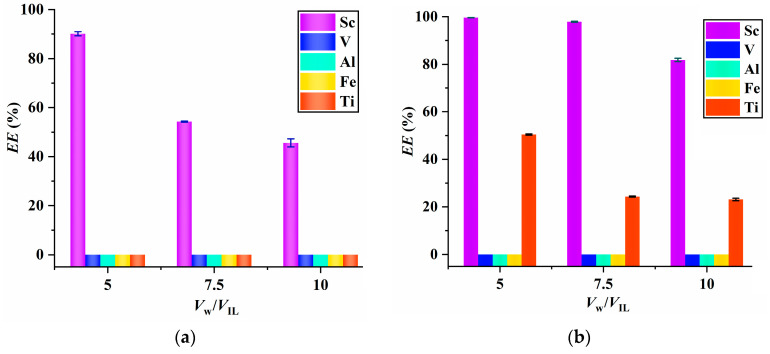
The *EE* values of [C_8_mim] NTf_2_/PPAH system toward Sc, Fe, V, Al and Ti in TDWA (**a**) and the leaching solution of red mud (**b**).

To investigate the applicability of the proposed extraction system, extraction experiments were conducted using [C_8_mim]NTf_2_/PPAH to extract Sc from real samples, TDWA and the leaching solution of red mud. The results displayed in Figure 6a indicate that the *EE* values of [C_8_mim]NTf_2_/PPAH toward Sc exceed 90% at a phase volume ratio of 5:1 and the *EE* values of Fe, V, Al and Ti are found to be zero, indicating the strong extraction selectivity of the [C_8_mim]NTf_2_/PPAH system. After extraction, the IL phase was dissolved with ethanol to generate a white precipitate (Sc(PPA)_3_) containing 9.4% of Sc (close to the theoretical value of 9.6%), along with small percentages of V (0.74%), Al (0.21%), Fe (0.84%) and Ti (0.056%), showcasing the high purity of the resulting Sc(PPA)_3_. Figure 6b demonstrates that for the extraction of Sc^3+^ in the leaching solution of red mud, the *EE* values of Sc^3+^ are above 97%, with a phase volume ratio ranging from 5:1 to 7.5:1. The *EE* values of Fe, V, Al are all equal to zero and the *EE* values of Ti decrease as the phase volume ratio increases. A phase volume ratio of 7.5:1 is a better choice, taking into account the *EE* value of Sc^3+^ and the extraction selectivity. The final product contains 0.21% of Sc (which is much lower than the theoretical value (9.6%)), 0.12% of Al, 16.5% of Ti, 1.6% of Fe and 0.0088% of V. This result suggests the poor purity of the final product, which may be related to the high content (530.8 mg L^−1^) of Ti in the leaching solution of red mud. How to purify the final product obtained from the leaching solution of red mud needs further study in future work.

Finally, to recycle the IL phase, a mixture of ethanol and [C_8_mim] NTf_2_ was distilled to eliminate ethanol, allowing the recovered [C_8_mim] NTf_2_ to be utilized in the subsequent extraction cycle.

## 3. Materials and Methods

### 3.1. Materials

Scandium chloride hexahydrate (ScCl_3_·6H_2_O, 99.9%), Scandium sulfate hydrate (Sc_2_(SO_4_)_3_·8H_2_O, 99.99%), D2EHPA (99%), TBP (≥99%), Nile Red (NR, ≥95%) and PPAH (≥98%) were purchased from Aladdin Biochemical Technology Co., Ltd. (Shanghai, China). Arsenazo Ⅲ (>95%) was obtained from Macklin Biochemical Technology Co., Ltd. (Shanghai, China). The ILs, 1-hexyl-3-methylimidazolium bis(trifluoromethylsulfonyl)imide ([C_6_mim]NTf_2_), 1-hexyl-3-methylimidazolium perchlorate ([C_6_mim]ClO_4_), 1-octyl-3-methylimidazolium bis(trifluoromethylsulfonyl)imide ([C_8_mim]NTf_2_), 1-octyl-3-methylimidazolium perchlorate ([C_8_mim]ClO_4_), 1-decyl-3-methylimidazolium bis(trifluoromethylsulfonyl)imide ([C_10_mim]NTf_2_) and 1-decyl-3-methylimidazolium perchlorate ([C_10_mim]ClO_4_), were supplied by the Lanzhou Institute of Chemical Physics, the Chinese Academy of Sciences (Lanzhou, China). 1-Butyl-3-octylimidazolium perchlorate ([C_4_C_8_im]ClO_4_), 1-butyl-3-octylimidazolium bis(trifluoromethanesulphonyl)imide ([C_4_C_8_im]NTf_2_), 1-butyl-3-decylimidazolium perchlorate ([C_4_C_10_im]ClO_4_) and 1-butyl-3-decylimidazolium bis(trifluoromethanesulphonyl)imide ([C_4_C_10_im]NTf_2_) were prepared via the methods reported in our previous work [34]. All other chemicals were of analytical grade unless otherwise stated. The TDWA sample (pH 0.36) containing 1.9 g L^−1^ of vanadium (V), 8.6 g L^−1^ of aluminum (Al), 16.6 mg L^−1^ of titanium (Ti), 82.6 g L^−1^ of iron (Fe) and 97.9 mg L^−1^ of Sc were supplied by a local titanium pigment plant using the chloride route. The dried red mud powder (about 100 mesh) was supplied by a local aluminum plant and its leaching solution was prepared via the reported method [48,49]: red mud powder was mixed with 1.0 mol L^−1^ of H_2_SO_4_ under stirring at 95 °C for 3 h with a liquid–solid ratio of 10:1 mL/g. After filtration, the resultant leaching solution (pH 0.91) containing 2.4 mg L^−1^ of Sc, 530.8 mg L^−1^ of Ti, 7.8 g L^−1^ of Al, 930.7 mg L^−1^ of Fe and 24.2 mg L^−1^ of V (determined by inductively coupled plasma optical emission spectrometry (ICP-OES, model Optima 8000, Perkin Elmer, Waltham, MA, USA) was used for subsequent extraction experiments.

### 3.2. Determination of the Polarities and Viscosities of the ILs

The polarities of the ILs used in this work were measured using solvatochromic dye and NR and defined as the molar transition energies of NR (*E*_NR_) [28,29,30]:

*E*_NR_ = 28,591/λ_max_ (11)

where λ_max_ represents the wavelength of maximum absorption of NR dissolved in a specific IL (NR concentration in each IL, 1.4 × 10^−5^ mol L^−1^) and this is measured by an ultraviolet–visible (UV-Vis) absorption spectrophotometer (model TU-1810, Purkinje General Instrument Co., Beijing, China).

### 3.3. Determination of the Solubilities of PPAH in ILs

To measure the solubilities of PPAH in ILs, excessive PPAH was mixed with a specific IL under stirring for 72 h at 298 K to assure the attainment of dissolution equilibrium and then this mixture was centrifuged for 2 min at 10,000 rpm to remove the undissolved PPAH. The supernatant was diluted by ethanol and the PPA content was then analyzed by the UV-Vis absorption spectrophotometer at 271 nm.

### 3.4. Extraction Procedure

For a typical extraction process, 0.5 mL of a specific IL containing PPAH was mixed with 10 mL of Sc solution under stirring at 298 K for 10 min. Following centrifugation, the Sc concentrations in standard solutions were measured by the visible spectrophotometry: 0.7 mL of arsenazo Ⅲ aqueous solution (0.5 g L^−1^), 0.75 mL of Sc solution (20 mg L^−1^), 1 mL of ethanol and 7.55 mL of monochloroacetic acid–NaOH buffer (0.2 mol L^−1^, pH 2.0) were mixed, the absorbance of the resultant mixture was measured at 660 nm and the Sc concentrations were calculated using the Beer–Lambert law. Furthermore, ICP-OES was used to measure the PPAH concentrations (obtained from the contents of phosphorus) in the water phases, the Sc contents in TDWA and the red mud leaching samples. The extraction efficiency (*EE*) was calculated using the following equation:

*EE*% = (1 − *C*_w_/*C*_w_^0^) × 100 (12)

where *C*_w_^0^ and *C*_w_ refer to the Sc concentrations in the aqueous phase before and after extraction, respectively.

### 3.5. Determination of the Stability Constant of Sc (PPA)_3_

To determine the stability constant (K) of Sc(PPA)_3_, an excess amount of Sc(PPA)_3_ powder was mixed with deionized water for at least 24 h to guarantee the achievement of dissolution equilibrium. The concentrations of Sc^3+^ and PPA^−^ were measured by the ICP-OES, as described above. The K value was calculated by the Equation (3) mentioned above.

All the above experiments were carried out in triplicate and the data reported in this work are expressed as means and standard deviations (SDs).

## 4. Conclusions

An IL-based extraction system, [C_8_mim]NTf_2_/PPAH was developed for the isolation of Sc from water phase and the experiments revealed that the *EE* values of Sc decreased as the alkyl side chain of the IL cation increased to decyl due to the formation of large-size nonpolar domains. Thermodynamic experiments suggested that the extraction efficiency of [C_8_mim]NTf_2_/PPAH toward Sc was over 96% in the temperature range of 288 K to 318 K and the extraction process of Sc was found to be endothermic. The physico-chemical properties of ILs including viscosity, polarity and hydrophobicity had minimal impact on the extraction of Sc. The proposed [C_8_mim]NTf_2_/PPAH system exhibited high selectivity toward Sc when it was applied to the isolation of Sc from TDWA but was not suitable for recovering Sc from red mud leachate. These findings suggested that the developed IL-based extraction method could be a promising approach for selectively recovering Sc from TDWA.

## Figures and Tables

**Table 1:** The water solubilities, viscosities and polarity of the ILs and the PPAH solubilities of the ILs used in this work.

IL	Water Solubility (g L^−1^)	Viscosity (mPa s)	Polarity (*E*_NR_, kcal mol^−1^) ^d^	PPAH Solubility in IL (g L^−1^) ^d^
[C_6_mim]ClO_4_	32 ^a^	107 ^a^	51.7	27.5
[C_6_mim]NTf_2_	2.6 ^b^	50.8 ^b^	52.2	12.0
[C_8_mim]ClO_4_	9.2 ^a^	170 ^a^	51.9	24.3
[C_8_mim]NTf_2_	1.0 ^b^	70.1 ^b^	52.3	12.5
[C_10_mim]ClO_4_	2.4 ^c^	363 ^d^	52.1	22.4
[C_10_mim]NTf_2_	0.7 ^b^	72.0 ^b^	52.2	10.0
[C_4_C_8_im]ClO_4_	3.2 ^e^	333 ^d^	52.0	21.6
[C_4_C_8_im]NTf_2_	0.31 ^e^	44.2 ^d^	52.2	11.4
[C_4_C_10_im]ClO_4_	0.93 ^e^	479.3 ^d^	52.0	21.5
[C_4_C_10_im]NTf_2_	0.086 ^e^	47.7 ^d^	52.4	12.3

^a^ Reference [31]; ^b^ reference [32]; ^c^ reference [33]; ^d^ this work; ^e^ reference [34].

## Data Availability

The data presented in this study are available in the article.
